# Outpatient Treatment of Refractory Morel-Lavallée Lesion Infection With Retention Sutures: A Case Report

**DOI:** 10.7759/cureus.42415

**Published:** 2023-07-25

**Authors:** Koji Miura

**Affiliations:** 1 Emergency Medicine, Kurashiki Central Hospital, Kurashiki, JPN

**Keywords:** vacuum assisted closure (vac), lower extremity trauma, vacuum-assisted closure, retention sutures, morel-lavallée lesion

## Abstract

We report the case of a 41-year-old man with a Morel-Lavallée lesion (MLL) that developed an infection. The patient was initially treated with intravenous antibiotics, but the infection persisted. He was then treated with outpatient cleansing and retention sutures, which resulted in successful wound healing.

This case report demonstrates the efficacy of outpatient cleansing and retention sutures for the treatment of MLL infection. This treatment modality is less expensive than inpatient treatment, and it allows the patient to return to their normal activities sooner.

## Introduction

A Morel-Lavallée lesion (MLL) is a subcutaneous dehiscence associated with high-energy trauma. MLLs have a high perioperative infection rate due to fluid retention in a closed cavity with limited blood flow. Once infected, they are difficult to treat, resulting in a prolonged hospital stay and a significant patient burden [1,2].

In this study, we report a case of recurrent infection of an MLL caused by a fall from a racing bicycle. The patient was initially treated with surgical drainage and irrigation, but the infection recurred. The patient was then treated with direct closure of the wound without a skin graft after outpatient cleansing with retention sutures, which successfully resolved the infection and shortened the hospital stay.

## Case presentation

A 41-year-old man was brought to our clinic after falling while riding a road bike alone. His vital signs were normal upon arrival, and he was alert and oriented. However, he had no memory of the fall. Physical examination revealed mild abrasions to the left iliac crest and left greater trochanter and abrasions to both knees. FAST (Facial drooping, Arm weakness, Speech difficulties, and Time) was negative, but CT imaging revealed a traumatic subarachnoid hemorrhage, a left 6-7 rib fracture, a mild left pneumothorax, and a left clavicle fracture.

The patient was admitted to the hospital for neurological monitoring and the fractures were managed conservatively. The symptoms did not worsen, and the patient was discharged on day 7.

Twelve days after the injury, the patient stood at home and experienced sudden pain and swelling in the lateral aspect of his left thigh. The patient had no fever, but the pain gradually worsened. He was admitted to the hospital on the fourteenth day after the injury.

**Figure 1:**
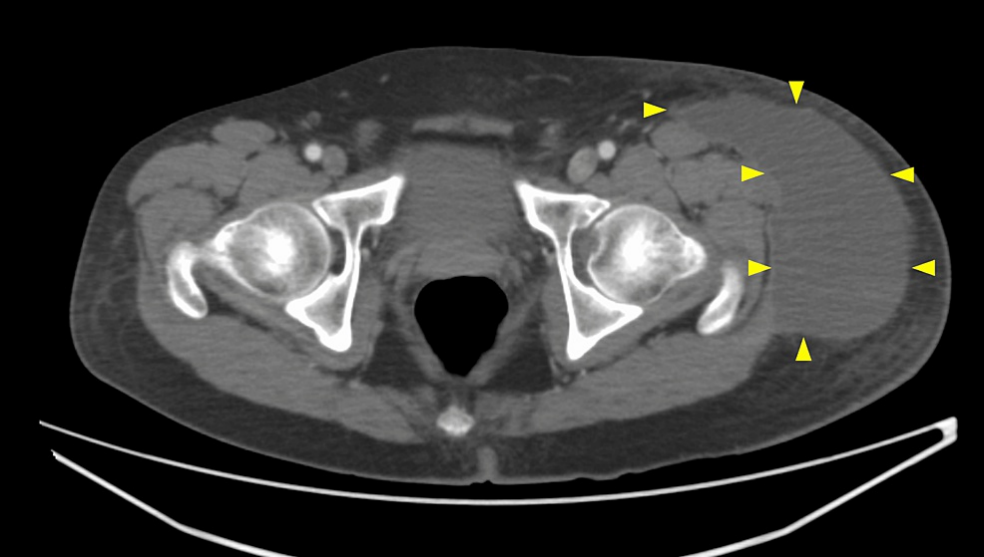
A CT image axial plane A hematoma-like effusion was observed on the lateral aspect of the iliotibial band (yellow arrowheads)

**Figure 2:**
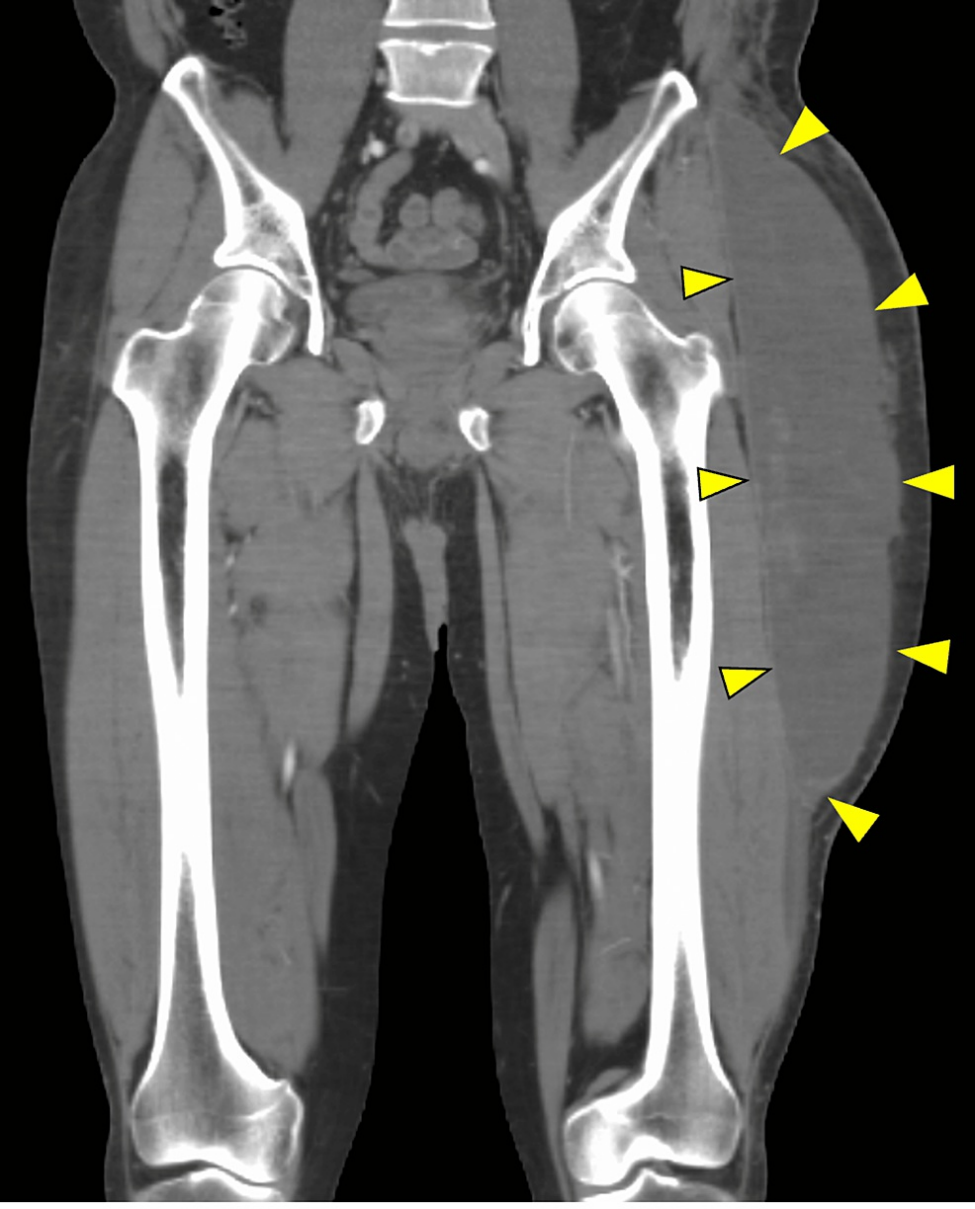
A CT image coronal plane A hematoma-like effusion was observed on the lateral aspect of the iliotibial band (yellow arrowheads)

On presentation, he had a fever of 38°C (100°F) and pain in the lateral thigh. The surface abrasion was healed. Ultrasonography revealed fluid collection in the subcutaneous tissue of the painful area. A CT scan confirmed a hematoma-like effusion on the lateral aspect of the iliotibial band (Figures 1, 2).

**Figure 3:**
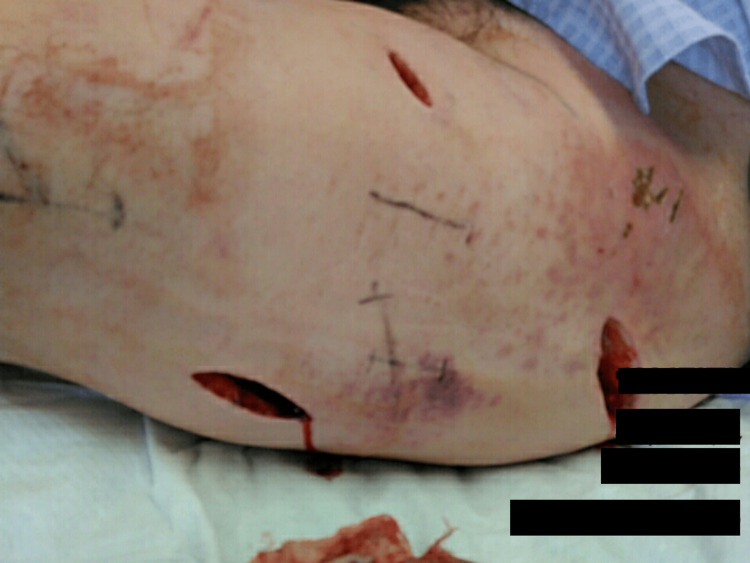
Skin incisions Three skin incisions were made for drainage and irrigation on the lateral aspect of the iliotibial band (head - right side and feet - left side)

Three skin incisions were made for drainage and irrigation (Figure 3).

The contents were pale, bloody, odorless, and measured 1200 mL. Due to a fever, the cyst was considered infectious, and vancomycin (VCM) + ceftriaxone (CTRX) was started. The wound was cleaned daily. Later, a drainage culture confirmed methicillin-sensitive Staphylococcus aureus (MSSA), and the antibiotic was changed to cefazolin (CEZ). 

There was no fever thereafter, and CT images confirmed that the abscess cavity had disappeared; therefore, the patient was discharged on day 29 after switching to oral antibacterial medication. On day 44 after the injury, outpatient follow-up confirmed no abnormalities in the wound or blood data, and antimicrobial therapy was discontinued.

**Figure 4:**
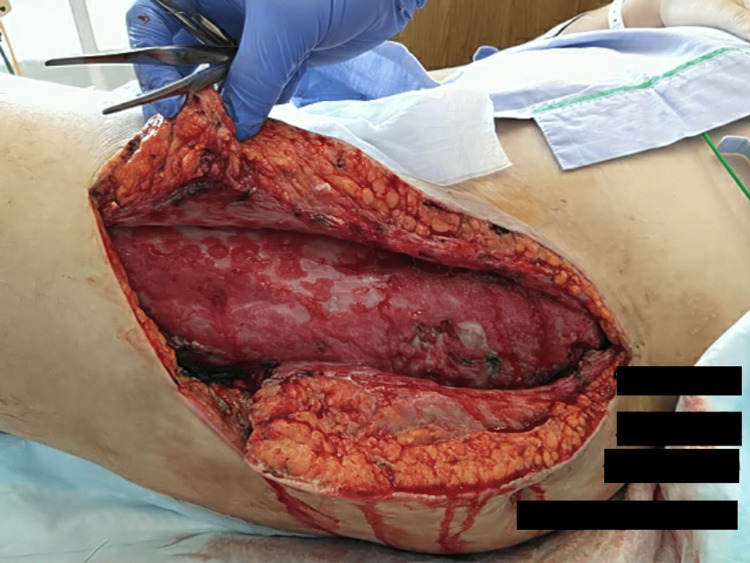
Opened abscess cavity The abscess cavity was surgically opened from top to bottom (head - right side and feet - left side)

On day 72, the patient presented to the hospital with lateral thigh pain and a fever of 39°C (102°F). A CT scan revealed fluid collection, and despite incisional drainage, he was unable to remain in the hospital. Instead, he was sent home with Keflex medication and was readmitted on day 79. Upon readmission, immediate surgical intervention involved the complete opening of the abscess cavity from top to bottom (Figure 4).

**Figure 5:**
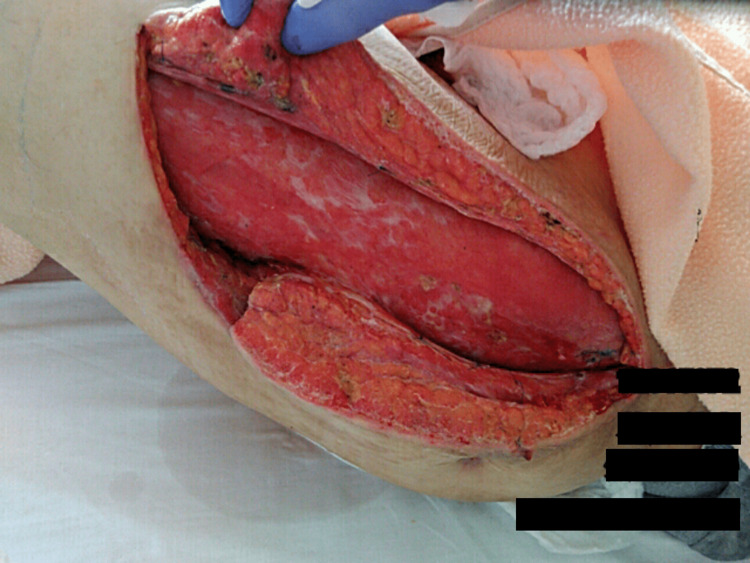
One week after open drainage The surface of the wound was clean (head - right side and feet - left side)

VCM was started due to the presence of methicillin-resistant Staphylococcus aureus (MRSA) in the previous culture. After one week of open drainage, vacuum-assisted closure (VAC) was initiated (Figure 5).

**Figure 6:**
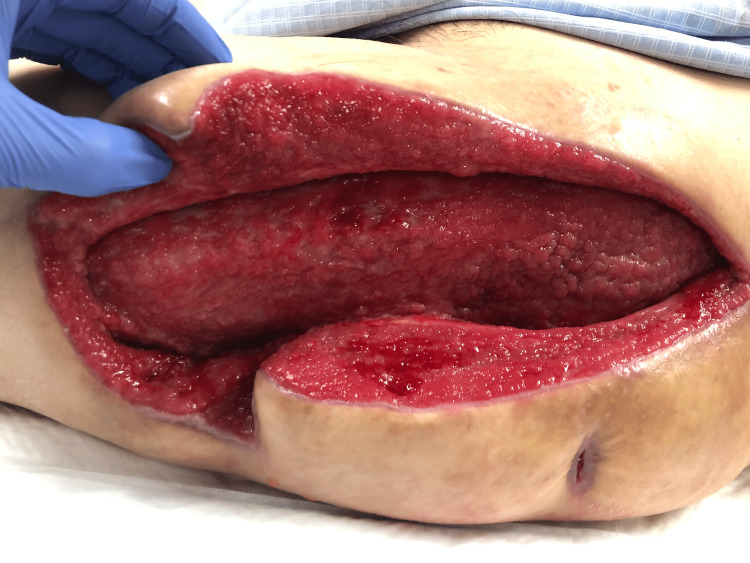
Recurrence of local infections One week after the VAC, due to the recurrence of local infections, we changed the treatment to open irrigation (head - right side and feet - left side). VAC: vacuum-assisted closure

However, one week later, due to the recurrence of local infections during VAC therapy, we changed the treatment to open irrigation (Figure 6).

**Figure 7:**
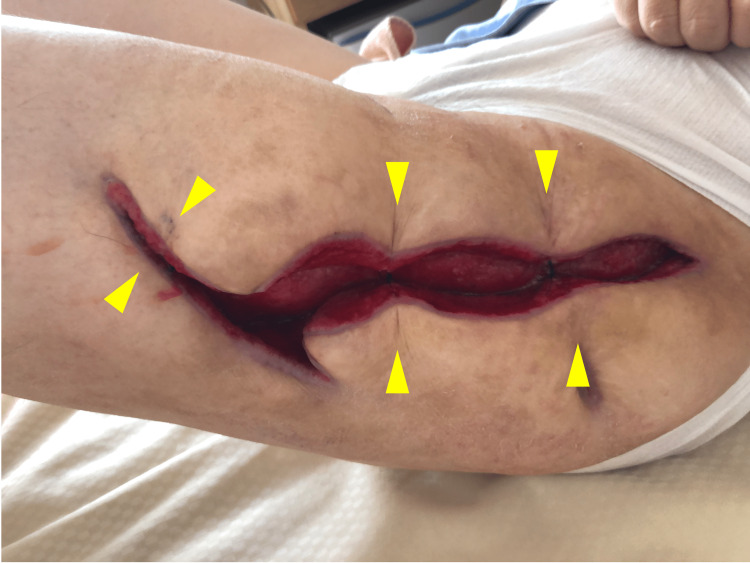
Three retention sutures on the wound To prevent skin regression, three retention sutures were placed (yellow arrowheads) (head - right side and feet - left side)

Therefore, after two weeks of open washing, retention sutures were placed with PDS II size 0 to prevent skin regression. Only three retention sutures were placed, leaving enough space at the wound margin to allow further wound washing (Figure 7). 

By this time, the patient's improved physical condition and the lessening of his wound pain made him feel distressed about his continued hospitalization, and he began to strongly desire discharge from the hospital for work. I reiterated the risk of treating the infection at home, but he no longer complied with it.

After receiving education on self-irrigation, the patient was discharged home with scheduled weekly outpatient wound monitoring. During this period, the ruptured PDS was reapplied as necessary. The self-irrigation process involved showering with copious amounts of tap water in the morning and evening.

Following a month of diligent home irrigation, the patient underwent wound closure due to the absence of infection and normal blood counts.

The surgical procedure was performed under lumbar anesthesia, during which the entire wound surface was carefully scraped with a sharp spoon. Subsequently, two 15 French negative pressure drains were placed after thorough irrigation with saline.

As perioperative antimicrobials, VCM was used against MRSA detected in previous cultures.

**Figure 8:**
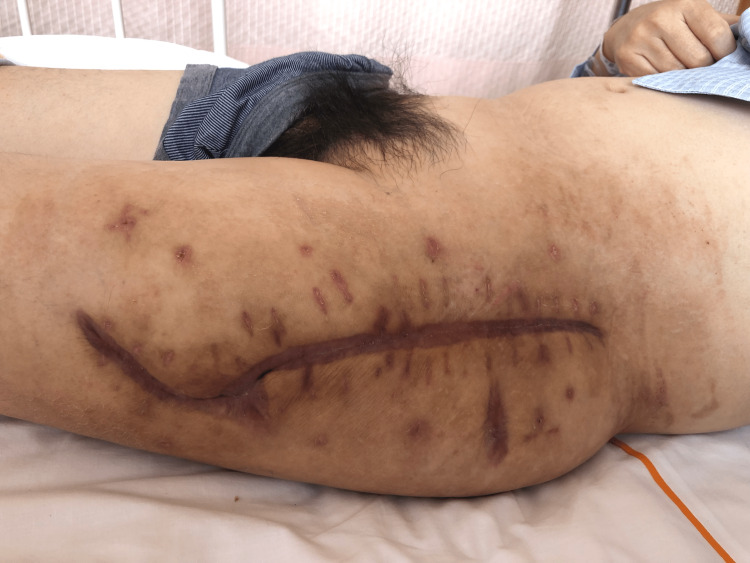
Final photograph of the closed wound POD 23 after completion of antimicrobial therapy. Wound closure was completed (head - right side and feet - left side). POD: postoperative day

Two weeks after surgery, the drains were successfully removed after confirming that the drain output had decreased to less than 30 ml per day [3]. However, two days after drain removal, local signs of infection were observed around the wound, leading to the initiation of CTRX to target the Escherichia coli detected in the drain tip culture at the time of removal. With the addition of CTRX, the patient showed improvement in local infection signs and blood counts returned to normal, eventually enabling discharge home on POD 23 after completion of antimicrobial therapy (Figure 8).

The patient was hospitalized for a total of 70 days and received antimicrobial therapy for 93 days. Three drainage procedures and multiple debridement procedures were performed.

One year has passed, and there has been no recurrence of the infection.

## Discussion

MLLs are difficult to treat when infection occurs because of fluid retention between the soft tissues and poor blood flow.

Previous reports have shown that the infection rate increases over time even when the wound is closed [1,2]. Early intervention is therefore advisable [4]. However, there are currently no reports on the use of various treatment modalities, including observation, puncture aspiration, surgical drainage, drain placement, open surgical lavage, or negative pressure closure [2,4-6].

Although the patient was a healthy young man with no underlying medical condition, he required prolonged treatment. Prolonged hospitalization causes economic and social hardships for patients. Therefore, there was a strong desire for outpatient treatment if possible.

Open lavage is the most effective treatment for persistent wound infections [7]. However, direct closure can be difficult later because of epidermal retraction over time [8]. VAC therapy is an effective means of treating these conditions; however, when wound closure makes it difficult to control infections, the use of retaining sutures can effectively clean the infected wound without closure and prevent epidermal regression.

In this case, two threads of PDS II (size 0) were used for each suture to provide adequate strength. The wound was approximately 25 cm long, and retention sutures were placed at three locations. Outpatient visits were made every week and retention sutures were reapplied as needed.

Consequently, the wound was closed without skin grafting. We believe that this was an effective method for extensive wounds refractory to wound infection, where VAC therapy was not feasible.

## Conclusions

This case demonstrated the efficacy of retention sutures as a treatment option for infected MLL. Retention sutures provide a valuable approach to achieving direct closure by effectively irrigating the wound and preventing skin regression.

In addition, the outpatient nature of this treatment option provides a significant advantage by reducing the emotional and financial burden associated with prolonged hospitalization, thereby improving the overall patient experience.
